# Quantifying, Understanding and Enhancing Relational Continuity of Care (QUERCC): a mixed-methods protocol

**DOI:** 10.1136/bmjopen-2024-088573

**Published:** 2025-04-29

**Authors:** Tom Marshall, Fiona Scheibl, Iestyn Williams, Krishnarajah Nirantharakumar, Brian H Willis, Panagiotis Kasteridis, Kamil Sterniczuk, Jinyang Chen, Zecharias Fetene Anteneh, Sheila Greenfield

**Affiliations:** 1 School of Health Sciences, Public Health and Epidemiology, College of Medicine and Health, University of Birmingham, Birmingham, UK; 2 School of Health Sciences, College of Medicine and Health, University of Birmingham, Birmingham, UK; 3 School of Social Policy, College of Social Sciences, University of Birmingham, Birmingham, UK; 4 Centre for Health Economics, University of York, York, Yorkshire, UK

**Keywords:** Patient Care Management, Person-Centered Care, HEALTH ECONOMICS, Quality in health care

## Abstract

**Abstract:**

**Introduction:**

Relational continuity of care is where patients see the same clinicians over time. Evidence suggests relational continuity of care is valued by patients and clinicians and results in better health. While current National Health Service policy aims to maintain relational continuity of care, it has been declining in recent years, which may be linked to the growth in practice size, increased staff turnover, part-time working and the focus on patient access. Our research aims to develop resources to help clinicians measure, manage and improve relational continuity of care.

**Methods and analysis:**

A mixed-methods approach in UK primary care commencing with two workshops drawing patients, clinicians and researchers together to establish an agreed approach on the measurement of continuity of care. Second, analysis of national data will provide insight into how staff turnover, part time working, practice size and funding per patient affects continuity. Third, case studies in a sample of high-performing practices will document the barriers and facilitators to the establishment and maintenance of continuity of care. Fourth, an economic analysis of resource costs and health outcomes using linked primary and secondary care data will show whether costs influence continuity for different patient groups (by age, sex, deprivation status and chronic disease status). Fifth, we will develop practical guidance for clinicians to improve continuity of care, based on the findings from each stage of the research.

**Ethics and dissemination:**

The study has approval from HRA Health and Care Research Wales Research Ethics Committee (HCRW). Findings will be disseminated through peer-reviewed publications, participatory workshops, podcasts, clinical networks and academic conferences.

**STRENGTHS AND LIMITATIONS OF THIS STUDY**

- This mixed-methods study engages a breadth of stakeholders in the development of resources aimed at understanding the context and measurement of relational continuity in primary care.
- A key benefit of the study design is the involvement of patients in the wider debate around the measurement and definition of relational continuity of care.
- Engagement with stakeholders is maintained throughout the study to maximise the relevance, quality and dissemination of study findings.
- Only general practices and stakeholders in England will be included in the study.

## Introduction

### Background

 Continuity of care includes informational continuity, sharing information between clinicians and organisations; management continuity, following the same management plan across different clinicians and organisations; and relational continuity, an ongoing therapeutic relationship between clinician and patient.^1^ Relational continuity of care (RCC) enables and underpins informational and management continuity and is important in primary care for two main reasons: it is valued by patients and general practitioners (GPs), and it is associated with better healthcare delivery and better health outcomes.^2^ Doctors and patients value RCC as facilitating the conditions required for person-centred care^3^,^6^ and view its absence as increasing the risk of harm and loss of trust.^27^,^9^ Long-standing evidence shows RCC is associated with reduced emergency consultations, unplanned admissions and even mortality^510^,^17^ across a range of acute and long-term conditions.^18^,^23^ Disruption of continuity is associated with increased use of specialty, urgent and emergency care in older patients.^24 25^ Furthermore, RCC is often particularly important in the delivery of primary care to diverse populations^6 26^ and can result in better care navigation and engagement among young people.^27^

However, continuity is declining. Lower continuity is associated with more clinicians working part time, use of locums, the growth in practice size with greater numbers of clinicians in practices and patient turnover.^28 29^ There is also an increased focus on patient access, rather than continuity. We currently do not know the extent to which practice-level characteristics undermine continuity or the extent to which continuity might be maintained or enhanced by within-practice policies. There are potentially many ways to optimise continuity. As no two general practices are the same, the most successful approach is likely to depend on the practice context. the Royal College of General Practitioners (RCGP) emphasises the need to measure relational continuity as a first step in its management.^30^

Very few general practices monitor the impact of their within-practice policies on continuity of care and conceptions of how to measure it differ. There is a long-recognised need for consistent measures of RCC.^5 31 32^ However, choosing an appropriate measure is complex.^33^ Subjective measures based on patients’ experience of continuity using questionnaires are impractical for monitoring.^34^,^37^ Measurement of the frequency of consultation with the same clinician is feasible using electronic health records (EHRs) and correlates with subjective measures.^38^ But different objective measures capture different conceptions of RCC.^31^ Continuity may be with the GP or with any clinician; it may be in all patients or in specific patient groups (eg, ≥65 years); it may be measured quarterly, monthly or weekly. There are different RCC indices. Some measure density: the Usual Provider of Care index (UPC) % of consultations with most frequently seen GP or the St Leonard’s Index of Continuity of Care (SLICC) % of consultations with a named GP.^39^ Others measure dispersion, taking account of the number of different clinicians consulted, using the Bice–Boxerman (BB) or Herfindahl (HI) indices. There is also a measure of Sequential Continuity (SECON) (table 1).

**Table 1:** Main indices of relational continuity of care

Name	What is measured	Formula
Usual Provider of Care (UPC^Patient^)	Concentration with usual provider	$max\left(\frac{n_{i}}{n}\right)$
St Leonards Continuity of Care (SLICC or UPC^GP level^)	Concentration with named provider	$namedclinician\left(\frac{n_{i}}{n}\right)$
Herfindahl Index (HI)	Concentration taking into account all providers	$\sum_{i=1}^{p}{\left(\frac{n_{i}}{n}\right)}^{2}$
Bice–Boxerman (BB)	Concentration accounting for the number of consultations	$\frac{\left(\sum_{i=1}^{p}n_{i}^{2}\right)-n}{n\left(n-1\right)}$
Sequential (SECON)	Sequential aspect of continuity	$\frac{\left(\sum_{j=1}^{n-1}c_{j}\right)}{\left(n-1\right)}$
Modified-Modified Continuity Index (MMCI)	Dispersion (lack of concentration)	$\frac{1-\frac{p}{n+0.1}}{1-\frac{1}{n+0.1}}$

*c_j_*, indicator of sequential visits to same providers, equal to 1 if visits *j* and *j*+1 are to the same provider, 0 otherwise; *n*, total number of visits during episode; *n_i_*, number of visits to provider *i*; *p*, total number of providers (clinicians).

Research has explored the effects of regularity and minimum frequency of contact on patients with chronic conditions.^40 41^ For patients who consult infrequently, measured continuity is arithmetically high; therefore, measured continuity declines with consultation frequency. Some RCC measures (eg, the BB) account for this. In practice, BB, HI, UPC and SECON are often highly correlated.^42^ SLICC is easy to calculate at the practice level and does not require patients to have a minimum number of consultations, but it may differ from the UPC if the patient’s usual GP and named GPs differ.^43^ A realistic strategy to improve continuity would also need to consider for which patient group it needs to be prioritised as the optimum balance between access and continuity may vary across different patient groups.

Given the complexity of the issues at stake, the Quantifying, Understanding and Enhancing Relational Continuity of Care (QUERCC) study aims to develop a menu of approaches to measuring RCC and empirically informed practical guidance to help general practices optimise it. QUERCC uses a mixed-methods design across five work packages (WPs) with defined objectives (table 2) leading towards project outputs and dissemination. To ensure QUERCC outputs are impactful, the team will work closely with the RCGP and international colleagues leading research addressing the same issues in different contexts.^44^

**Table 2:** Objectives of the Quantifying, Understanding and Enhancing Relational Continuity of Care study

Objectives	Work package	Outcome
Develop guidance for general practices on quantitative measurement of RCC.	WP1	A menu of approaches to measuring RCC for monitoring in primary care.
Quantify the practice-level determinants of RCC: including staff and patient turnover, part time working, practice size and practice funding. Identify practices showing unusual variation (positive deviants) in RCC.	WP2	A model of the contribution of patient and general practice-level characteristics to trends and variations in RCC. An observed to predicted ratio of RCC in Clinical Practice Research Datalink practices. A list of general practices in the top decile for RCC will be abstracted and sites selected for inclusion as potential case studies.
Conduct in-depth case studies to understand how practices achieve high RCC.	WP3	The primary output is an understanding of the practice characteristics which contribute to RCC and understanding of barriers and facilitators to RCC and the mechanisms by which RCC influences health.
Undertake economic analysis of the likely causal effects of changing RCC on resource costs and health outcomes across different segments of the registered practice population.	WP4	A model assessing the impact of changes in RCC on healthcare resources and health outcomes. The model will describe the effects of changing RCC on different population groups.
Develop empirically informed practical guidance to help general practices optimise RCC.	WP5	Develop guidance deriving from existing research and stakeholder input to produce in-depth empirical data on the strategies for increasing and/or maintaining RCC in primary care settings. We will document the full range of these strategies and interventions and provide a summary of evidence on their efficacy and implementation in different settings. These outputs will be a key part of our dissemination and knowledge exchange activities and will therefore inform practice in the immediate term. We will also identify areas where there might be a need for intervention *adaptation* or de novo.

RCC, relational continuity of care.

## Methods and analysis

### Research design

**Figure 1:**
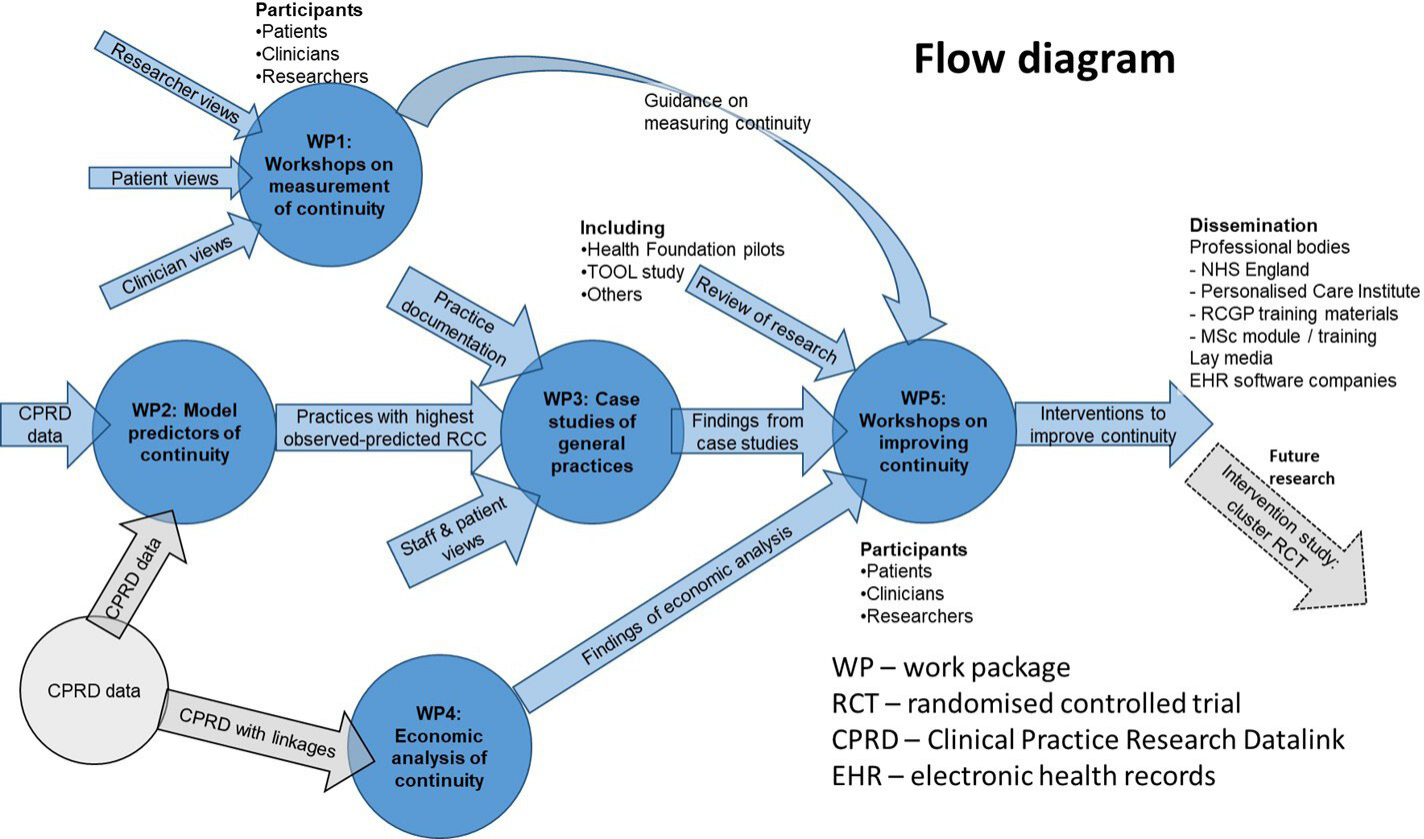
Quantifying, Understanding and Enhancing Relational Continuity of Care work packages and description of data sets, data collection and analysis.NHS, National Health Service; RCGP, Royal College of General Practitioners.

**Figure 2:**
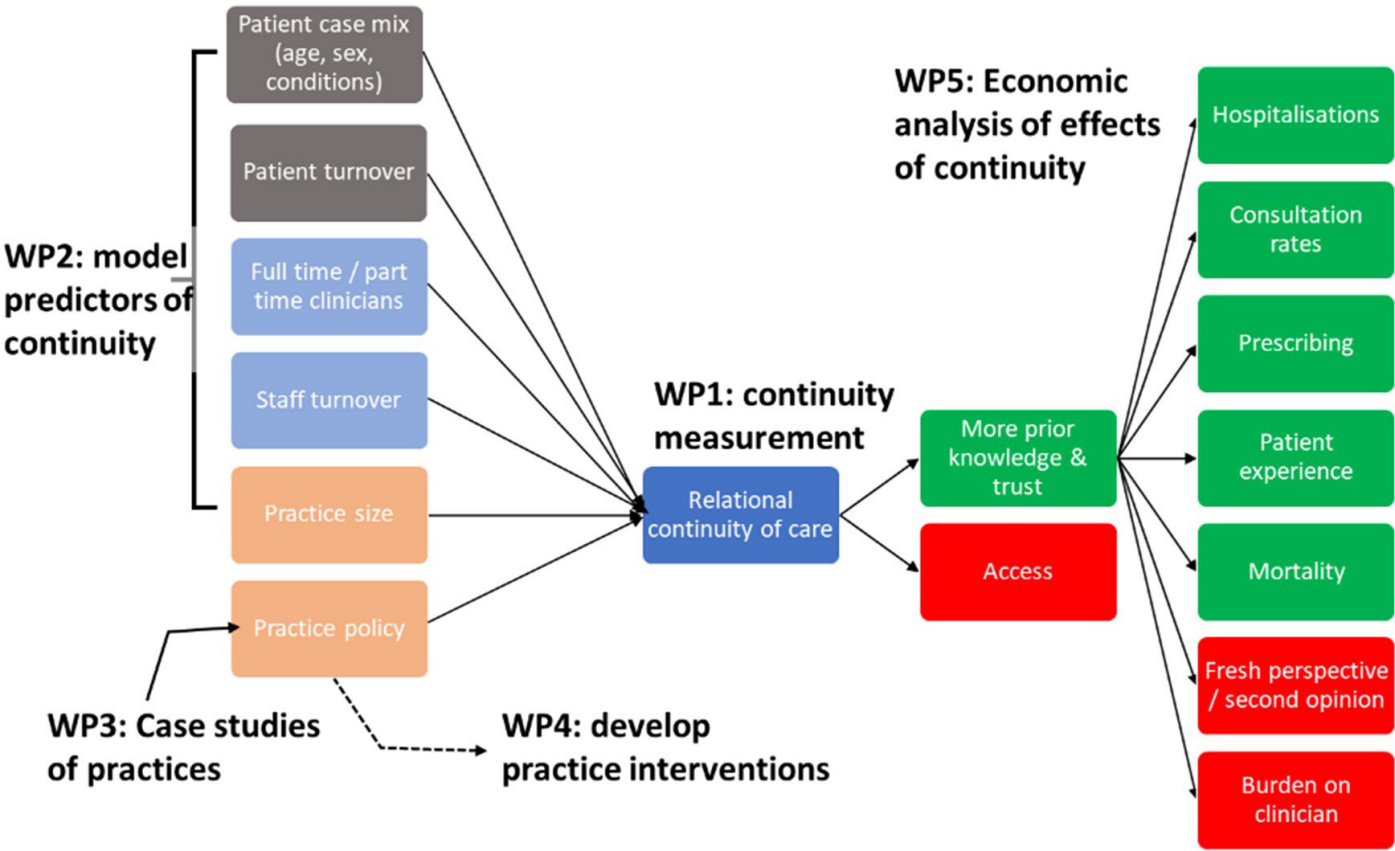
Work packages (WPs) addressing the core determinants of and effects of relational continuity of care.

A mixed-methods approach is used to capture the complexities of continuity of care as a measurable event with associated outcomes and subjective experience and an organisational process and value. Our approach is designed to develop empirically informed strategies for improving and maintaining RCC in primary care settings(see figure 1). The study will commence in April 2023 and conclude in May 2026. WPs 1–3 will draw together insights from consensus workshops (WP1), case studies (WP3) and Clinical Practice Research Datalink (CPRD) data (WP2) to identify factors that explain the practice-level drivers of continuity of care (see figure 2). These findings will be integrated with the economic evaluation (WP4), leading to the development of a menu of approaches to measuring RCC, and empirically informed practical guidance to help general practices optimise it (WP5).

Data collection across the five WPs addresses the possible determinants of RCC (patient characteristics, patient and staff turnover, part-time and full-time working, practice size and within-practice policies) and its effects, including both positive effects (on hospitalisations, consultation rates, prescribing, patient experience and mortality) and negative effects (lack of a second opinion, clinician burden). Consideration is also given to the interplay between RCC and access to primary care.

### Ethics and dissemination

Ethical approval has been obtained from the Health Research Authority (HRA) and Health and Care Research Wales Research Ethics Committee (23/SW/0101 and 24/EM/0031). We will share research findings accross a range of academic publications, networks and conferences.

### Funding statement

This work was supported by the National Institute of Health Research [NIHR] under the Health and Social Care Delivery programme [HSDR]. Reference: NIHR152277 QUERCC. HSDR funding call: 22/16 HSDR May 2022.

### Data statement

Technical Appendix, including topic guides and qualitative data set, will be made available at OSF | Quantifying, Understanding and Enhancing Relational Continuity of Care (QUERCC). Quantitative data set and models are available from the authors.

### Patient and public involvement

During the conception and development of this proposal, we consulted with eight patients in two workshops. Participants had lived experience of RCC becoming more difficult to maintain and something that they valued in healthcare. They identified more doctors working part time, larger practices, automated booking systems and receptionists as potential barriers to continuity. These issues are also reported in the literature and informed our decision to conduct a mixed-methods programme of research. We established a Patient Advisory Group (PAG) (five members) in October 2023 and recruited three patient and public involvement (PPI) representatives in January 2024. The study PPI lead/co-applicant (KS) came through to work on the project from the original cohort consulted in 2019. The PAG and PPI have contributed to patient-facing documents developed for WP1 and WP3 and worked together to shape the content of the study website. PAG and PPI will contribute to data review across all five WPs, working with the team to maximise knowledge sharing. The PPI lead will attend monthly management meetings, and three PPI members will have optional attendance to contribute to the management of the project.

### Theoretical framework

Synthesis and interpretation of data collected across the five WPs will be guided by the recently updated Consolidated Framework for Implementation Research (CFIR).^45^ CFIR is one of the most widely used frameworks to organise and interpret data on factors that shape implementation of change within healthcare settings.^45 46^ It provides a framework of 39 implementation constructs which facilitates the organisation and interpretation of data across five core domains: outer setting (eg, the economic, political and social context), inner setting (eg, the structural, political and cultural context where the implementation takes place, such as an organisation), characteristics of individuals (eg, attitudes, values and beliefs of the individuals involved) and process (eg, components that impact the implementation process). Working in this way the analysis will aim to unpack the key components of a workable intervention.

### WP1: identifying areas of divergence and consensus in the measurement of RCC in two qualitative consensus workshops

Using coproduction and consensus methods across two workshops, this WP aims to determine how clinicians, patients and researchers define continuity and which RCC measures they recognise as offering the best intuitive approach to its measurement. This WP opens a stakeholder debate around the development of guidance for practices and the choice of RCC index to adopt and in which populations to measure RCC which will feed into all five WPs.

#### Selection of workshop participants

Clinicians, patients and researchers will be recruited using purposive sampling methods. We aim to recruit through local patient networks to sample a range of patients by age, gender, ethnicity, education level and where possible chronic disease status. Professionals will be identified and recruited through national and regional professional networks who will be selected to represent a diversity of age, gender and ethnicity. Working in this way we aim to recruit a minimum of 15 and a maximum of 30 participants with equal numbers across the patient and professional categories.

#### Data collection

Data are collected face to face in two workshops convened in central Birmingham and professionally facilitated. All participants will be consented to take part and reimbursed for their time at appropriate NHS locum rates (clinicians) and rates set by NIHR guidelines (lay participants) (Payment guidance for researchers and professionals | NIHR).

#### Approach

In workshop 1, participants will be invited to contribute three key ideas on what RCC means to them and given time ahead of the workshop to write these ideas down. Once in the workshop, a professional facilitator skilled in coproduction approaches and the theme lead will work with participants in three breakout groups to select a key idea to bring to a wider group discussion. During the group discussion, the theme lead will explain the breadth of issues in RCC measurement and participants will be asked to individually reflect on the key components of RCC (eg, ‘Is continuity primarily with one GP, more than one GP or all clinicians?’ ‘What aspects of care are most important in providing continuity?’). The discussion and breakout group ideas will be charted visually, and key ideas generated in the debate summarised under thematic headings. Participants will be invited to vote on key thematic headings built on the ideas they contributed. The facilitator will use coproduction methods to work towards a consensual perspective that respects the range and depth of the stakeholder themes generated. Coproduction approaches will enable us to bridge any discursive gap between lay and medical understandings of RCC.

In workshop 2, which will be held approximately 2 months later, we will work with the same participants (or substitutes if some cannot attend) and professional facilitator to examine the issue of RCC measurement. The team lead will generate a series of visual scenarios and outline the pros and cons (see table 3) of currently used indices (BB, HI, UPC^Patient^ or UPC^GP level^).^47 48^ We will record the discussion to aid analysis and thematic summary.

**Table 3:** Illustration of pros and cons of different measures of relational continuity of care

			Continuity index				
**Clinician group**	**Patient group**	**Criterion**	**SLICC**	**UPC**	**BB**	**Hi**	**SECON**
Consultation with GP only	Age ages	Understandable	Good	Good	Fair	Fair	Fair
		Unaffected by consultation rate	Poor	Poor	Good	Good	Fair
		Patient conception	Fair	Fair	Good	Good	Poor
		Clinician conception	Fair	Fair	Fair	Fair	Fair
	Aged 65+ only	Understandable					
		Unaffected by consultation rate					
		Patient conception					
		Clinician conception					
Consultation with all clinical staff	All ages	Understandable					
		Unaffected by consultation rate					
		Patient conception					
		Clinician conception					
	Aged 65+ only	Understandable					
		Unaffected by consultation rate					
		Patient conception					
		Clinician conception					

BB, Bice–Boxerman index; HI, Herfindahl index; SECON, Sequential Continuity; SLICC, St Leonard’s Index of Continuity of Care; UPC, Usual Provider of Care index.

Facilitated group discussion will examine key problems involved in the measurement of RCC (eg, ‘Is continuity primarily with one GP, more than one GP or all clinicians?’ ‘What aspects of care are most important in providing continuity?’ ‘How frequently should it be measured?’). Stakeholders will be invited to vote on a range of measures of RCC (eg, which population, density vs dispersion, GPs or all clinicians, understandability, etc). It is anticipated that voting will be supported with an online voting tool (Interactive presentation software - Mentimeter) to gauge support for different measures presented.

#### Data analysis

A manifest content analysis approach will be used to examine the overlap and divergence of views on the definition of continuity of care obtained in workshop 1. Summary analysis will identify the points of convergence and difference across the participant groups (clinicians, researchers and patients). Transcripts and voting results from workshop 2 will be summarised in an Excel spreadsheet, and further content analysis will summarise the range of perspectives on measurement and the value of a range of RCC indexes.

#### Output

We do not anticipate that the workshop data will generate a complete consensus on a single way to measure RCC but expect these stakeholder conversations to inform and sensitise analysis and modelling in WP2 and subsequent WPs.

### WP2: investigation of determinants of relational continuity of care

The aim of this WP is to investigate practice-level determinants of measured RCC in general practices and identify practices showing unusually high continuity given their characteristics for inclusion as case studies in WP 3. We will examine how RCC is related to the practice population’s characteristics (age, sex, ethnicity, chronic disease status, deprivation) and practice characteristics (practice size, patient turnover, clinician turnover, workload, part time working, funding levels).

#### Data and methods

We will investigate the determinants of RCC using CPRD data; a large primary care database linked to data on practice funding. CPRD collects fully coded and de-identified patient EHRs from a network of GP practices using the Vision (CPRD GOLD) or EMIS (CPRD Aurum) software systems (further details provided in online supplementary material). CPRD data are broadly representative of the English general population.^49^ We will use data from the CPRD GOLD database for the period 1 January 2005 until the most recent data upload linked to the General and Personal Medical Services database (NHS Digital) from which we will obtain data on average funding per registered patient (further details provided in online supplementary material). Modelling will enable us to calculate a predicted monthly RCC for each participating general practice; from this, we can calculate an observed to predicted ratio of RCC. Unusual variation in a process is more likely to have an assignable cause.^50^ Therefore, it is likely to be productive to investigate outlier practices as case studies. To shortlist potential case studies in the most recent quartile of RCC data, we will identify the top decile of general practices by their observed to predicted ratio of RCC. We will use a range of measures to predict the ratio of RCC: UPC, HI, BB, Modified Modified Continuity Index and the SECON Index. Prior analysis shows that these measures report very similar lists of positive deviants. We will ultimately use the measure that best predicts clinical costs and outcomes. The measure used may not be the same measure preferred by stakeholders (clinicians, patients and researchers) in the workshops in WP1. This reflects the pragmatic requirement that the WP2 model is specified to incorporate costs and clinical outcomes.

#### Output

A model of the contribution of patient and general practice characteristics to trends and variations in RCC. An observed to expected ratio of RCC in CPRD practices. Identification of general practices in the top decile for RCC for inclusion as potential case studies in WP3.

### WP3: qualitative case study of the determinants of continuity of care

In WP3, we will use an exploratory multiple case study design sampling positive deviant cases to capture a rich description of processes and interactions in practices.^51^ Previous research suggests that selecting case studies from outliers (deviant cases) is a valid way to find out about causal pathways and causes of heterogeneity.^52^ For example, a similar method has been used to investigate wards providing safe hospital care.^52 53^

#### Sample selection

Deviant ‘outlier’ sites for WP3 case study will be identified during analysis of CPRD data completed in WP2. We will identify a quartile (180) with the highest continuity of care (RCC) and a quartile (180) with average RCC. Within these quartiles, we will also consider the size of practices with high and low RCC and whether the populations they serve are more, or less, deprived. We aim to sample eight ‘deviant’ sites: six general practices from the quartile with the highest continuity of care (from the top 10%) and two general practices from those with average continuity of care (the lowest 10%).

#### Site recruitment

CPRD data are anonymised, which means it is not possible to identify organisations prior to obtaining their agreement to take part as case studies. To facilitate recruitment, we will enlist the CPRD agency to send invites to practices that meet the inclusion criteria on behalf of the QUERCC study. We will supply CPRD with a template letter to send out. Recruitment will be ongoing until eight sites agree to take part. To maximise recruitment rates, we will facilitate participation by offering flexible times and either hybrid or in- person options for data collection.

#### Participant recruitment to focus groups and interviews at case study sites

We will recruit 15–30 participants at each of the eight case study sites to take part in two focus groups: one with a range of clinical and non-clinical staff, and one with practice patient participation groups. Focus groups will be supplemented with semistructured interviews with up to three key informants per practice (identified during the focus groups). Interviews will enable the further investigation of themes across the organisational strata, including depth perspectives from reception and administration staff who may not be able to join the focus group. A depth patient interview will also add nuance to understandings and make provision for any patient who, for example, cannot make the focus group. This size and number of focus group are optimal for data collection in a case study context.^54 55^

We will aim to recruit a diversity of clinicians and patients by age, gender and ethnicity. We will also aim to select a range of patients by education level and chronic disease status and use an equality and diversity form to collect participants’ details anonymously. An interpreter will be made available to any potential participant whose first language is not English and wants to take part. We will translate the Patient Information Sheets/Equality and Diversity/Informed Consent Forms and other study documents on their behalf to enable them to fully consult the terms of the study and consider the implications of participation. We will use topic guides to structure the focus group discussion and interviews. Recruitment will exclude patients who lack capacity to consent and a lower age limit of >20 and an upper age limit of <95 years. The number of participants recruited and consented to take part will be in the region of n=72–216 (2 focus groups (6–12 people) + 3 interviews × 8 sites).

#### Data analysis and synthesis

Thematic and framework approaches^56^ will be used to work with qualitative data and integrate these with the findings of the quantitative data collected in WP2. Themes will be compared within and across practices, and across data collection method focus group, interview and documentary data.^57^ A summary of overall themes from their discussion will be sent to participants for comment. PPI representatives and members of the multidisciplinary research team (SG, sociologist; TM, public health clinician; IW, health service policy analyst) will read a selection of transcripts and documents, then discuss and agree on emerging themes to develop the data coding framework. To ensure robustness and quality, our research and analysis will also be guided by the Consolidated criteria for Reporting Qualitative research checklist for reporting qualitative research,^58^ and data will be ‘triangulated’ across the WP data sources across the study. Comparing and synthesising data in this way will provide additional insights and enhance understandings. Overall findings will then be brought together and considered by the whole research team.^59 60^

#### Output

The primary output of WP3 is an understanding of the practice characteristics which contribute to RCC and the barriers and facilitators to the provision of RCC. A secondary output will be to gain insight into the mechanisms by which RCC influences health.

### WP4: economic analysis of the effects of RCC

In this WP, we will analyse the potential effects on resource use and health outcomes of changes in practice-level RCC using patient-level data. We will analyse the effects across different segments of the registered practice population.

#### Data and methods

We will use primary care data from the CPRD database from 1 January 2005 until the most recent upload, with standard linkages to (1) Hospital Episode Statistics data (including inpatient admissions, outpatient appointments and A&E attendances), (2) Office for National Statistics mortality data and (3) area-level deprivation. We will also use one non-standard linkage to funding per patient. CPRD Gold includes about 9 million patients eligible for linkage in around 400 general practices and CPRD Aurum 38 million eligible patients in around 1400 general practices (Clinical Practice Research Datalink | CPRD).

We will analyse the effects of RCC on (1) two types of primary care use: consultations and prescribing; (2) three types of hospital use: unplanned admissions, A&E presentations and outpatient appointments; (3) costs and (4) mortality. We will identify primary care activity from the CPRD records of consultations, clinical events and prescription records and secondary care activity from Hospital Episode Statistics on inpatient, outpatient and A&E records. We will cost primary and secondary care activities using the methodology that we have previously used.^61^

We will undertake a patient-level analysis with the explanatory variable of interest (RCC) measured at practice level. We will undertake preliminary analyses to explore the relationship between RCC and unplanned admissions (the main driver of costs) over time. If the relationship was altered during the pandemic years, we will consider whether to consider prepandemic and postpandemic years separately. We will also undertake preliminary analyses to explore whether the relationship between RCC and unplanned admissions varies by chronic disease status (using chronic diseases included in the Quality and Outcomes Framework) to determine whether analysis should be segmented by chronic disease status.

The study population will consist of patients who were registered with a GP practice any time during the period from 1 January 2006 to 31 March 2021 (currently most linked data are available up to 2021). We will observe these patients until outcome or censoring, where censoring is due to the patient changing GP practice, death or the end of the study period (date of last upload). The observation period for each patient will be divided into periods of 3 months, and outcomes will be binary variables indicating whether or not the particular event occurred in each 3-month period (except from costs which are a continuous variable). For instance, a patient initially observed on 1 January 2017, who underwent inpatient hospitalisation in March 2018, will contribute data for five quarters: four quarters in 2017 where the outcome value is zero and one quarter in 2018. The resulting data set will be an unbalanced panel as individuals contribute to the sample of different number of quarters depending on when they experience an outcome. RCC will be measured at practice level over the 12 months prior to the outcome period using the indices from WP1 (eg, in the above example, the RCC associated with the patient’s first observation will be measured over the period 1 January 2016 to 31 December 2016). We will include a mix of patient-level confounders such as age, gender, deprivation, ethnicity, morbidity profile, prior healthcare utilisation and practice-level characteristics such as practice size, practice funding, staff turnover and part time working.

We will employ discrete-time survival analysis to evaluate the association between the risk of each outcome in a particular 3-month period and RCC in the prior 12 months. Specifically, we will estimate complementary log-log (cloglog) models (the discrete-time analogue of the continuous-time proportional hazards models) which are appropriate when the occurrence of an outcome is rare.

#### Output

A model assessing the impact of changes in RCC on healthcare resources and health outcomes. The model will help us understand the effects of changing RCC on different population segments.

### WP5: empirically informed practical guidance to help improve RCC in primary care

This final phase of this research is to collate and integrate findings from different WPs, generate and disseminate learning, and create impact. It involves co-designing principles and methodologies to develop guidance on how to improve and enhance RCC. We will develop empirically informed practical guidance to help general practices optimise RCC using a Normalisation Process Theory (NPT) framework relevant to primary care interventions^62 63^ and disseminate findings to stakeholders.

## Method: evidence review co-design and stakeholder engagement

We will undertake a rapid review of evidence on within-practice interventions^64^,^66^ making use of ongoing reviews on this topic.^67^ We will follow good practice and consider or ‘triangulate’ findings from the evidence review and this study’s WPs. We will follow good practice and consider or ‘triangulate’ findings from the evidence review and from our own study’s WPs: WP1 (meaning of RCC), WP2 (determinants of RCC), WP3 (detailed case studies) and preliminary results from WP4 (economic analysis). Each separate WP will have individual and stand-alone findings analysed separately using techniques appropriate to their methods, but we will additionally look at all of the different sets of findings together, to identify what each contributes to the overall picture. We will use principles of NPT to combine the findings from each WP. Comparing and contrasting individual WP findings against NPT is likely to provide additional insights and enhance overall understanding. Overall findings will then be brought together and considered by the whole research team. We will convene two 3-hour deliberative workshops (either face-to-face or online) to integrate these findings and develop practical guidance on how best to improve RCC. This process will be informed by existing research on how to ensure the needs of diverse groups are taken into consideration^68^ using coproduction methods^69^ to ensure that recommendations are acceptable to patients and clinicians and are deliverable.^70^

As with WP1, participants will include a purposive sample of clinical and non-clinical professionals and patient representatives. Participants who attended workshops in WP1 will be given the opportunity to take part, and if needed, we will invite additional attendees, drawing on networks and groups identified over the life course of the study.

The first workshop will be carried out with the patients only (n=~15) in order to determine their views. In this workshop, we will investigate what role patients and the public might have in facilitating RCC in different settings. We will explain the background to the project and share preliminary findings from our case studies on the characteristics of general practices associated with high RCC and the perspectives of staff and patients in these practices. Before the workshop, we will summarise the findings in plain language form for participants to read and will briefly present the findings at the start of the workshop. We will ask participants to reflect on the evidence in relation to their own experience to identify which characteristics might form the basis of practice policies which are acceptable to patients. We will also ask them to identify any knowledge gaps. To facilitate discussion, participants will be broken up into smaller groups (six or less). The headline conclusions of the workshop will be summarised at the end of the workshop to ensure these have been captured accurately. Notes and minutes of the workshop will be collated and summarised by the research team and circulated to workshop participants for their final approval.

In the second workshop, both patients and practice staff (n=~30) will meet together for joint discussion. We will again provide the findings in written form for participants to read before the workshop and briefly present findings at the start of the workshop, including key messages from the first workshop. We will ask participants to identify practice characteristics which might form the basis of acceptable and feasible practice policies on RCC. We will ask participants to achieve satisfactory agreement on the information content and medium of delivery of a final set of recommendations on optimising RCC. We will also ask participants to identify any knowledge gaps. To facilitate discussion, participants will be broken up into smaller groups (six or less). Workshops will be facilitated by a professional facilitator supported by the study team and audio-recorded, and audios will be deleted following verification of anonymised transcript. Transcripts will be analysed using the principles of framework analysis with the specific purpose of informing the development of good practice principles to support RCC.^71^ The research team will then draft a written document with recommendations and circulate this to participants for final comment.

## Ethics and Dissemination

The main ethical considerations in this study relate to participant anonymity. Data collection approaches are designed to ensure informed consent and the safeguarding of personnal data which will be stored in line with institutional policies. We will provide potential particpants with information about the study and time to consider their participation. Interview participants will not be named or identifiable, and we will use pseudonyms to report any direct quotes.

For a lay audience we will create a project website and commission a short animation of our final project report to communicate findings in an accessible way. The project website will make available regular publicly accessible bulletins of interest to the general public. We will initate a social media campaign to promote interest, communicate findings and encourage feedback via blogs. Through our digital profile, we will engage with patient groups and third-sector organisations as intermediaries and knowledge brokers to help us develop an effective implementation and dissemination strategy and to ensure we engage heterogeneous groups of stakeholders. We plan to share our code for measurement of RCC with manufacturers of primary care records software (initial discussions have been advanced with developers at Cededim and clinical computer system developed by the Horsforth-based The Phoenix Partnership) to stimulate and facilitate the development of tools to measure RCC. To directly reach primary care clinicians, we will develop a podcast or short video with the Personalised Care Institute to disseminate to clinicians through the development of two webinars and a package of marketing and communications with the RCGP. The University of Birmingham’s Centre for Primary Care Improvement will create a postgraduate module for primary care professionals on managing continuity of care.

### Declaration of Helsinki

This study complies with the Declaration of Helsinki, adopted by the 18th World Medical Association (WMA) General Assembly, Helsinki, Finland, June 1964 and last revised by the 64th WMA General Assembly, Fortaleza, Brazil, October (2013).

## Supplementary material

10.1136/bmjopen-2024-088573 online supplemental file 1
