# Supplementary material for: Quantifying, Understanding and Enhancing Relational Continuity of Care (QUERCC): a mixed-methods protocol
Source: BMJ Open. 2025 Apr 29;15(4):e088573. doi: 10.1136/bmjopen-2024-088573 (PMC12049910; doi:10.1136/bmjopen-2024-088573)
Supplement: online supplemental file 1 [file bmjopen-15-4-s001.docx]

#### **WP2 Investigation of determinants of relational continuity of care & identification of outliers**

Research suggests RCC is influenced by the practice population’s characteristics (age, sex and chronic disease status) and practice characteristics (practice size, patient turnover, clinician turnover, part-time working) and may be influenced by practice funding levels. We will investigate the determinants of RCC through analysis of a large primary care database, linked to data on practice funding and subjectively reported continuity.

**Aim:** to investigate patient and practice-level determinants of measured RCC in general practices and to identify practices showing unusually high continuity given their characteristics.

**Method:** Clinical Practice Research Datalink (CPRD) collects fully coded and de-identified patient electronic health records from a network of GP practices using the Vision® (CPRD GOLD) or EMIS® (CPRD Aurum) software systems. We have full access to both datasets. CPRD includes records of clinical events (medical diagnoses), referrals to specialists and secondary care settings, prescriptions issued in primary care, records of immunisations/vaccinations, diagnostic testing, and all other types of care administered as part of routine general practice. Clinical information is captured as hierarchical Read codes, which are recorded by practice staff as part of routine data entry. CPRD data are broadly representative of the English general population.^[[1]](#endnote-1)^ We will use data from the CPRD GOLD database for the period 1^st^ January 2005 until the most recent data upload, with two additional linkages. One is to GPPS data (2007 onwards), to allow comparison between subjectively reported continuity to objectively and measured RCC. The other is to the General and Personal Medical Services database (NHS Digital), to obtain data on average funding per registered patient.

We will measure monthly RCC outcome at the level of general practice using the continuity index most strongly associated with health outcomes and resource use. The chosen index may differ to indices prioritised in WP1, which are selected to reflect staff and patient preferences and are for internal practice use. To assess its construct validity, we will correlate practice-level measured RCC with annual subjectively reported RCC from the GPPS. Candidate predictors of RCC include patient characteristics and practice-level characteristics, all measured monthly. Patient characteristics are mean age (a), percentage female (f_%_), prevalence of chronic disease (CD_%_) and prevalence of multimorbidity (M_%_). Chronic disease prevalence is the proportion of patients with one or more of the chronic diseases in the Quality and Outcomes Framework (QOF) and multimorbidity is the proportion of patients with ≥4 chronic diseases. We already have code lists for over 80 chronic conditions including those in QOF. Practice list size (L) is based on monthly counts and patient turnover (P_T_) from monthly registration and deregistration data. We will determine total number of GPs (GP_n_) in the practice month from the numbers with attributable consultations in that month. We will infer a GP has left when they have no consultations for >13-weeks and use this to calculate a monthly turnover rate of GPs (GP_t_). We will do the same for non-GP clinicians if our RCC index requires it. We will infer part-time working from the usual pattern of weekday consultation over each month (consultations morning and afternoon on 5 days = full time) and calculate an appropriate summary measure of part-time working: percentage full time equivalent (FTE_%_). If available we will include linked data on practice funding per registered patient (F_£_).

We expect to have roughly 15 years-worth of data, from 1800 general practices. Using monthly summaries will provide 180 (15×12 observations) for each practice. We will model the association between monthly RCC outcome and candidate predictors (listed above and including both patient and practice characteristics, all measured at the practice-level: a, f_%_, CD_%_, M_%_, L, P_T_, GP_n_, GP_t_, FTE_%_, F_£_) using regression analysis. To examine the independent contributions of these characteristics to RCC, the regression analysis will include all ten candidate predictors of interest in the model regardless of statistical significance - no stepwise variable selection will be used - as is recommended.^[[2]](#endnote-2)^ Using monthly practice summary statistics for each candidate predictor will ensure there will be no missing data in either candidate predictors or RCC outcome. Monthly practice RCC outcomes are expected to be correlated - with outcomes measured close in calendar time expected to be more highly correlated than those measured further apart in calendar time. To allow for this serial correlation all regression analysis will allow for auto-correlations. The exact form of these correlations will be dependent on the data, but we will explore various forms, such as auto-regressive structures as well as allowing for seasonality, using recommended approaches to identify best fitting structures.^[[3]](#endnote-3)^ The contribution of the ten candidate predictors will be modelled to allow for non-linear effects, and without categorising any continuous predictors.^[[4]](#endnote-4)^ Again, appropriate forms to model non-linear effects will be explored and are likely to include splines or fractional polynomials. To internally validate the model all coefficients and standard errors (including associated confidence intervals) will use bootstrap-based confidence intervals with shrinkage. The ability of the model to correctly identify high performing RCC practices will be summarised using calibration and discrimination statistics (again using appropriate shrinkage to avoid over fitting).

It has long been recognised that unusual variation in a process is more likely to have an assignable cause.^[[5]](#endnote-5)^ Evidence from analysis and simulation shows that selecting case studies from outliers (deviant cases) is an efficient way to find out about causal pathways and causes of heterogeneity.^[[6]](#endnote-6)^ We will identify potential case studies from the top decile of general practices over a period of one year. In the most recent year’s data, we will use the derived prediction model to identify practices which, given their patient and practice characteristics, appear to be consistent high performers in RCC. Using the derived model, we will calculate a predicted monthly RCC for each participating general practice for each month. We can then calculate an observed to predicted ratio of RCC. These monthly practice level observed / predicted ratios averaged over the most recent year, ranked and practices in the top decile of these ratios identified.

**Output:** A model of the contribution of patient and general practice-level characteristics to trends and variations in RCC. An observed to predicted ratio of RCC in CPRD practices. A list of general practices in the top decile for RCC in the most recent data quartile for inclusion as potential case studies.

1. Herrett E et al. Data Resource Profile: Clinical Practice Research Datalink (CPRD). Int J Epidemiol. 2015 Jun;44(3):827-36. [↑](#endnote-ref-1)
2. Steyerberg, Ewout W., et al. Prognosis Research Strategy (PROGRESS) 3: prognostic model research. PLoS medicine 2013: 10.2: e1001381. [↑](#endnote-ref-2)
3. Turner SL et al. Evaluation of statistical methods used in the analysis of interrupted time series studies: a simulation study. BMC Med Res Methodol. 2021;21(1):181. doi: 10.1186/s12874-021-01364-0. [↑](#endnote-ref-3)
4. Royston P, Altman DG. Regression using fractional polynomials of continuous covariates: parsimonious parametric modelling. J R Stat Soc: Series C (Applied Statistics) 43.3 (1994): 429-453. [↑](#endnote-ref-4)
5. Shewhart WA. Economic control of quality of manufactured product. New York. 1931. [↑](#endnote-ref-5)
6. Seawright J. Multi-Method Social Science: Combining Qualitative and Quantitative Tools. Chapter 4 - Case Selection after Regression. Cambridge University Press 2016; 75-106. [↑](#endnote-ref-6)
