# Supplementary material for: Quantifying, Understanding and Enhancing Relational Continuity of Care (QUERCC): a mixed-methods protocol
Source: BMJ Open. 2025 Apr 29;15(4):e088573. doi: 10.1136/bmjopen-2024-088573 (PMC12049910; doi:10.1136/bmjopen-2024-088573)
Supplement: Supplementary file 2 [file bmjopen-2024-088573.reviewer_comments.pdf]

PEER REVIEW HISTORY

BMJ Open publishes all reviews undertaken for accepted manuscripts. Reviewers are asked to complete a checklist review form and are provided with free text boxes to elaborate on their assessment. These free text comments are reproduced below.

ARTICLE DETAILS

Title (Provisional)

Quantifying, Understanding and Enhancing Relational Continuity of Care (QUERCC) A mixed methods protocol.

Authors

Marshall, Tom; Scheibl, Fiona; Williams, Iestyn; Nirantharakumar, Krishnarajah; Willis, Brian H; Kasteridis, Panagiotis; Sterniczuk, Kamil; Chen, Jinyang; Anteneh , Zecharias Fetene; Greenfield, Sheila

---

VERSION 1 - REVIEW

**Reviewer**	**1**
**Name**	**Scholtes, Stefan**
**Affiliation**	**University of Cambridge, Centre for Health Leadership and Enterprise, Judge Business School**
**Date**	**19-Jul-2024**
**COI**	**N/A**

---

1. Just as a personal note: I am wondering whether you are unnecessarily complicating your message by using the qualifier "relational" continuity of care. It is diffiicult to actually measure the relational aspect. What is generally measured is the extent to which patients have consultations with the same or a small group of physicians over time. These measures will likely be highly correlated with all aspects of continuity of care (informational, management, accountability, etc). In other words, GPs will find it difficult to build relationships with their patients unless they have enough interactions with them - and these frequent interactions will also have other beneficial aspects.

2. There were minor copy-editing errors, e.g. "an ongoing between clinician and patient", “therapeutic relationship” missing.

**Reviewer**	**2**
**Name**	**Hawksworth, Olivia**
**Affiliation**	**The University of Sheffield, Clinical Trials Research Unit**

Date **03-Jan-2025**

COI **None**

Thank you for the opportunity to review this manuscript. Relational continuity of care is an interesting and timely area for study and the research has the potential to support improvements in relational continuity of care in GP practices.

There are some areas where additional clarification would be helpful for the reader:

1. It is not immediately clear what the participants will be voting on in consensus workshop 1 (“Participants will be invited to vote on key themes and the facilitator will work towards a consensual perspective...”). Are they voting on which aspects of continuity are most important to them?

2. An explanation of how the findings from WP1 about defining continuity and the important elements of continuity will inform the recommendations in WP5 would be beneficial.

3. In WP3, focus group discussions are supplemented by interviews. I think the purpose of these interviews should be described – what will they add beyond the focus groups?

4. One important consideration that is touched on is whether efforts to improve continuity should be directed towards all patients, or targeted for particular groups of patients who are likely to benefit most. Will this be considered in any of the work packages?

Minor comments:

5. In the description of WP1, you refer to the INVOLVE payment guidelines. It would be good if these were cited.

6. There is a spelling error on page 11 (“Excell” spreadsheet)

7. Much of the information about WP2 is included as supplementary information. It would be good to point to the supplementary material within the manuscript.

8. It would be useful to know which indices will be used to calculate monthly RCC in WP2. In the supplementary material it says that these might differ from those identified in WP1.

9. On page 13, continuity of care (CoC) is referred to, whereas in the rest of the paper relational continuity of care (RCC) is discussed. Was it specifically relational continuity that was calculated to determine the inclusion of sites as case studies? If so, then RCC should be used.

10. On page 16 line 23, there is a minor error in the wording “a patient…will contribute to the data five quarters”.

11. There is an error with the cross referencing on page 17.

12. In the output and dissemination paragraph, you say that you will “share findings on the measurement of RCC”. It might be beneficial to add a few words to explain what is meant by this.

VERSION 1 - AUTHOR RESPONSE

<table>
<tr><th>Item comment</th><th>Authors response</th></tr>
<tr><td>Reviewer: 1
Prof. Stefan Scholtes, University of Cambridge
Comments to the Author:
1. Just as a personal note: I am wondering whether you are unnecessarily complicating your message by using the qualifier "relational" continuity of care. It is difficult to actually measure the relational aspect. What is generally measured is the extent to which patients have consultations with the same or a small group of physicians over time. These measures will likely be highly correlated with all aspects of continuity of care (informational, management, accountability, etc). In other words, GPs will find it difficult to build relationships with their patients unless they have enough interactions with them - and these frequent interactions will also have other beneficial aspects.

2. There were minor copy-editing errors, e.g. "an ongoing between clinician and patient”, “therapeutic relationship” missing.</td><td>1. Thank you for highlighting this issue. The authors acknowledge the challenge of measuring the relational aspect of RCC. The aim of QUERCC is to advance the understanding of RCC. In WP1 we will work with stakeholders to establish a common or shared understanding of RCC. Findings from this phase will be used to evaluate existing measures. Working with the RCGP we aim to offer guidance on which measure does the best job of measurement to respect or encompass the relational aspect of continuity.

2. Thank you for noting the typos we have addressed these.</td></tr>
<tr><td>Reviewer: 2
Dr. Olivia Hawksworth, The University of Sheffield

There are some areas where additional clarification would be helpful for the reader:
1. It is not immediately clear what the participants will be voting on in consensus workshop 1 (“Participants will be invited to vote on key themes and the facilitator will work towards a consensual perspective...”). Are they voting on which aspects of continuity are most important to them?
2. An explanation of how the findings from WP1 about defining continuity and the important elements of continuity will inform the recommendations in WP5 would be beneficial.
3. In WP3, focus group discussions are supplemented by interviews. I think the purpose of these interviews should be described – what will they add beyond the focus groups?
4. One important consideration that is touched on is whether efforts to improve continuity should be directed towards all patients or targeted for particular groups of patients who are likely to benefit most. Will this be considered in any of the work packages?
Minor comments:
5. In the description of WP1, you refer to the INVOLVE payment guidelines. It would be good if these were cited.</td><td>Additional clarification for the reader:

1. We have explained more fully that stakeholders will vote on the different types of measures e.g. which population, density v dispersion, GPs or all clinicians, understandability etc) in workshop2. We have explained that stakeholders will vote on the themes they developed during workshop1.
2. We have explained we will use Normalisation Process Theory to combine the findings from all work packages.
3. We have added more detail about the interview process for WP3.
4. The issues highlighted will be addressed throughout the project but in particular WP1 and WP5 and considered as part of guidance issued.

Minor comments:
5. We have added a direct link to NIHR guidance in the text for ease of reference.</td></tr>
</table>

6. There is a spelling error on page 11 ("Excell" spreadsheet)<br>7. Much of the information about WP2 is included as supplementary information. It would be good to point to the supplementary material within the manuscript.<br>8. It would be useful to know which indices will be used to calculate monthly RCC in WP2. In the supplementary material it says that these might differ from those identified in WP1.<br>9. On page 13, continuity of care (CoC) is referred to, whereas in the rest of the paper relational continuity of care (RCC) is discussed. Was it specifically relational continuity that was calculated to determine the inclusion of sites as case studies? If so, then RCC should be used.<br>10. On page 16 line 23, there is a minor error in the wording "a patient…will contribute to the data five quarters".<br>11. There is an error with the cross referencing on page 17.<br>12. In the output and dissemination paragraph, you say that you will "share findings on the measurement of RCC". It might be beneficial to add a few words to explain what is meant by this.	6. Thank you we have corrected the spelling error.<br>7. We have inserted pointers to the supplementary material as requested.<br>8. We have added further details about the indices to be used.<br>9. Thank you for noting this error. The calculation is for RCC, and we have removed the term CoC and replaced it with RCC.<br>10. We have edited the sentence to clarify.<br>11. We have removed the field code reference which was not required and inserted in error.<br>We have added more details on the plan to share findings with software companies.

VERSION 2 - REVIEW

Reviewer 1

Name Scholtes, Stefan

Affiliation University of Cambridge, Centre for Health Leadership and Enterprise, Judge Business School

Date 07-Apr-2025

COI

Thank you for your response to my query.

Reviewer 2

Name Hawksworth, Olivia

Affiliation The University of Sheffield, Clinical Trials Research Unit

Date 01-Apr-2025

COI

Thank you for responding to my comments, I am happy that the points I raised have been addressed.
